# Supplementary material for: Influence of Fermentation Beetroot Juice Process on the Physico-Chemical Properties of Spray Dried Powder
Source: Molecules. 2022 Feb 2;27(3):1008. doi: 10.3390/molecules27031008 (PMC8840475; doi:10.3390/molecules27031008)
Supplement: Supplementary file 1 [file molecules-27-01008-s001.zip › molecules-1556312-supplementary.pdf]

Influence of fermentation beetroot juice process on the physico-chemical properties of spray dried powder

Emilia Janiszewska-Turak [1,*], Maciej Walczak [1], Katarzyna Rybak [1], Katarzyna Pobiega [2], Małgorzata Gniewosz[2], Łukasz Woźniak [3] and Dorota Witrowa-Rajchert

Table S1. Pre-tests results for juices

Sample Name	Bacteria count (log CFU/g)	Betalain content (mg/100g d.m.)	Vulgaxanthin-I content (mg/100g d.m.)
LB_3	6.61 ± 0.14	755.59±46.90	288.37±8.71
LB_4	7.09 ± 0.13	884.69±233.63	297.12±75.54
LB_5	7.13 ± 0.06	867.59±175.56	272.10±38.76
LB_6	7.21 ± 0.18	944.52±181.54	301.96±49.64
LF_3	6.25 ± 0.09	723.59±49.06	266.05±3.90
LF_4	7.22 ± 0.16	824.19±58.83	247.98±15.62
LF_5	7.25 ± 0.03	788.74±49.8	251.36±1.61
LF_6	7.34 ± 0.05	826.11±48.30	237.10±13.57
LP_3	6.45 ± 0.05	737.18±59.37	292.89±31.26
LP_4	7.39 ± 0.08	705.96±31.24	235.48±14.05
LP_5	7.33 ± 0.19	883.87±36.73	319.15±24.53
LP_6	7.47 ± 0.16	940.16±112.95	321.74±57.07

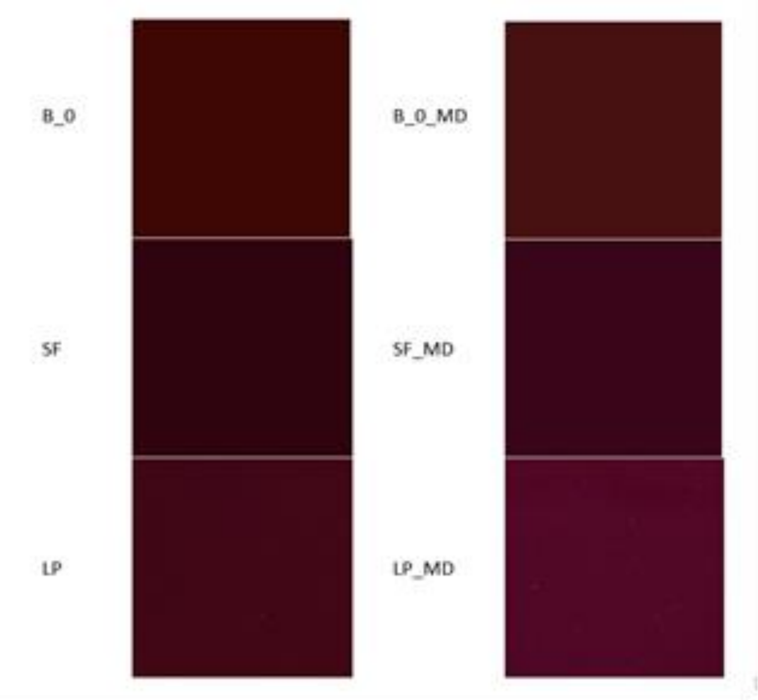

a)

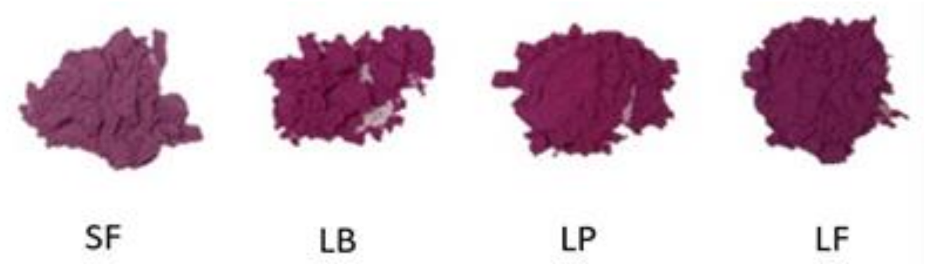

b)

Figure S1. Photo of: a) beetroot juice and fermented beetroot juice without and with carrier (Maltodextrin -MD); b) obtained powders * SF- *spontaneous fermentation*, LB - *Levilactobacillus brevis*, LF - *Limosilactobacillus fermentum*, LP - *Lactiplantibacillus plantarum*), SF_5_MD – juice from fifth day of spontaneous fermentation with 10% addition of MD

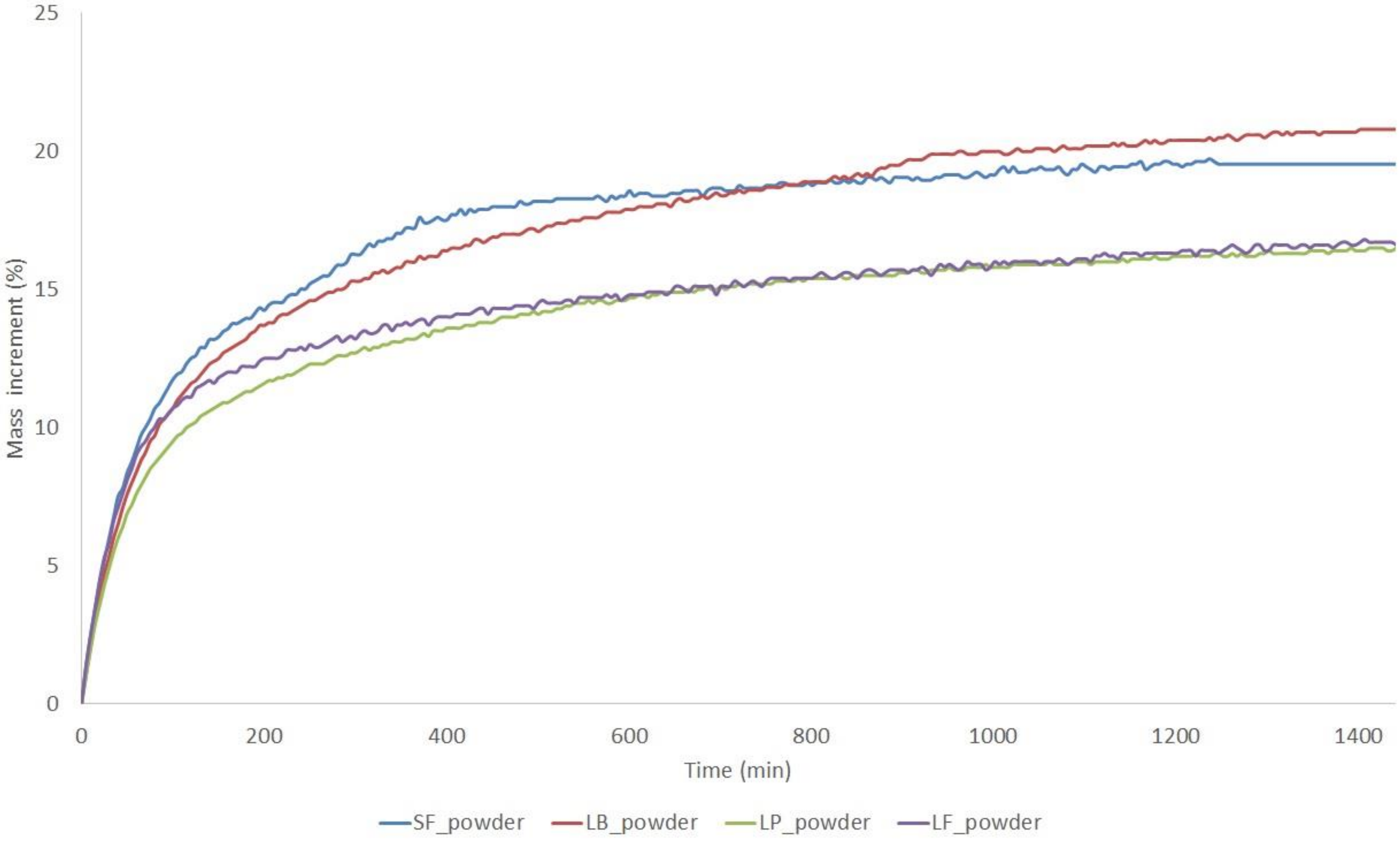

Figure S2. Sorption kinetics for powders (*SF- spontaneous fermentation, LB - *Levilactobacillus brevis*, LF - *Limosilactobacillus fermentum*, LP - *Lactiplantibacillus plantarum)*

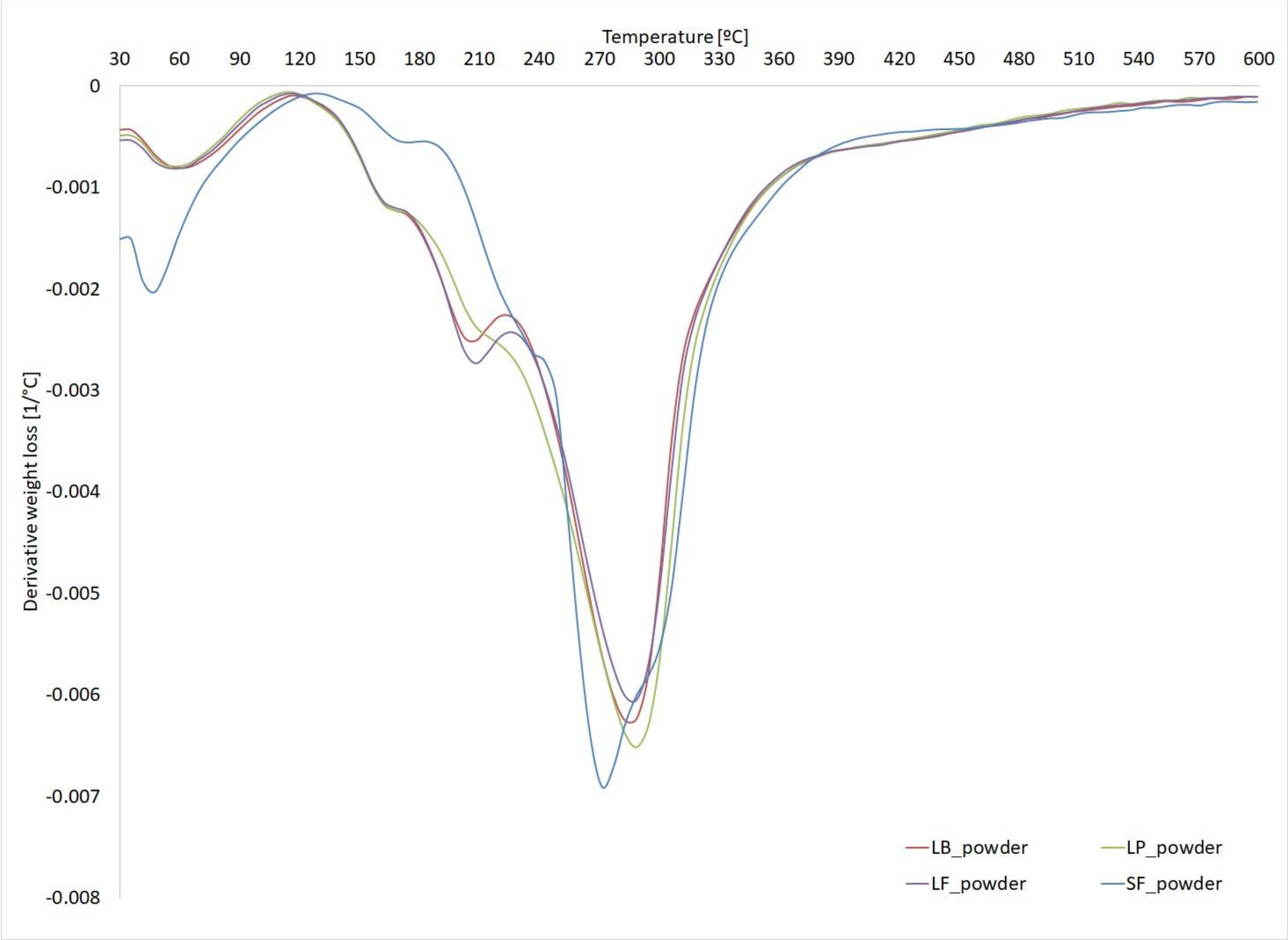

Figure S3. Derivative curves of TGA analysis of powders (*SF- spontaneous fermentation, LB - *Levilactobacillus brevis*, LF - *Limosilactobacillus fermentum*, LP - *Lactiplantibacillus plantarum)*
